# Mutational Characterization of Colorectal Cancer from Korean Patients with Targeted Sequencing

**DOI:** 10.7150/jca.61324

**Published:** 2021-10-28

**Authors:** Jongmin Lee, Sangtae Choi, Donghae Jung, YunJae Jung, Jung Ho Kim, Sungwon Jung, Won-Suk Lee

**Affiliations:** 1 Gachon Institute of Genome Medicine and Science, Gachon University Gil Medical Center, Incheon, Republic of Korea; 2 Gachon Advanced Institute for Health Science and Technology, Gachon University, Incheon, Republic of Korea; 3 Department of Surgery, Gachon University College of Medicine Gil Medical Center, Incheon, Republic of Korea; 4 Department of Pathology, Gachon University College of Medicine Gil Medical Center, Incheon, Republic of Korea; 5 Department of Microbiology, Gachon University College of Medicine, Incheon, Republic of Korea; 6 Department of Internal Medicine, Gachon University College of Medicine Gil Medical Center, Incheon, Republic of Korea; 7 Department of Genome Medicine and Science, Gachon University College of Medicine, Incheon, Republic of Korea

**Keywords:** Colorectal cancer, targeted sequencing, mutation, Korean population

## Abstract

**Purpose:** Effective treatment of colorectal cancer could benefit from understanding molecular characteristics including mutation profiles of important genes. This study aimed to explore the molecular characteristics of colorectal cancer based on next generation sequencing.

**Methods:** The mutational characteristics by targeted next generation sequencing in 172 colorectal tumor samples from Korean patients were evaluated to explore their associations with clinical features. Targeted sequencing of 375 genes was performed with an average target-depth of 800X.

**Results:** TP53 and APC showed higher mutation frequencies from the left-sided tumors, while CTNNB1 were more frequent from the right-sided tumors. The tumor suppressor NOTCH1 and the DNA strand break repair gene PALB2 were more frequently mutated in early onset tumors. KRAS and PTEN mutations were more frequent from patients with advanced cancers by cancer antigen markers. TP53 and BRAF mutations were more frequent from patients of T3 and T4 stages, where their variant allele fractions were generally higher in T4 tumors, implying that advanced tumors have higher fraction of cancer cells with TP53 and BRAF mutations. Mutational profiles of these patients were also assessed with other clinical features. Comparison of mutational characteristics with the Caucasian subjects from independent data showed that the identified mutational characteristics are largely Korean-specific except for a few key colorectal cancer genes.

**Conclusion:** Next generation sequencing-based targeted sequencing can provide valuable information on molecular characterization of colorectal cancer patients, and its clinically relevant information can provide benefits to better understand colorectal cancer.

## Introduction

The clinical impact of tumor location (i.e left VS. right sidedness) on patient survival and response to chemotherapies has been shown in large clinical trials 1. However, the underlying molecular biology explaining such differences has not been clearly defined. The anatomical difference can be considered from embryological origin. The right colon receives blood supply from superior mesenteric vessels, a midgut structures from the mid-duodenum to the mid-transverse colon, whereas the inferior mesenteric artery supplies hindgut structures from the mid-transverse colon to the rectum. Aside from this embryological point of difference, colon cancer has been recently subcategorized to the following CMS subtypes: 1) microsatellite instability immune (14%), 2) canonical (37%), 3) metabolic (13%), 4) mesenchyme (23%) and 5) mixed features (13%) 2. The differential distribution of the five classes in various anatomic regions suggests biological differences in the right-sided colon and left-sided colon cancer. It has been shown that right-sided colon cancer patients were older, had larger tumor sizes and more advanced tumor stages, were more often female, and had poorly differentiated tumors 1. Petrelli et al. 3 revealed in their large cohort study that left-sided colon cancer was associated with a significantly reduced risk of death, which was independent of stage, race, and adjuvant chemotherapy. However, questions still remain despite such huge clinical differences, mainly because molecular understanding is as yet scattered and not comprehensive. In this study, we investigated the genetic aberrations in clinical colon cancer samples to further delineate these molecular differences.

Multiple studies investigated the association between mutation frequencies and clinical characteristics including tumor locations from colorectal cancer. Lee et al. 4 compared the frequencies of somatic mutations between left-sided and right-sided tumors. Lieu et al. 5 investigated the differences in gene mutations with varying ages of patients. Shen et al. 6 evaluated the prognostic impact of different mutation profiles in stage II and III colorectal cancer patients. Poulos et al. 7 compared the mutation profiles across colorectal cancer patients with varying MSI status. Bordonaro and Lazarova 8 investigated the mutation profiles with varying obesity status of patients, and suggested association between obesity and lower mutation threshold. However, there has been limited study on the mutational and clinical characteristics of colorectal cancer from East Asia including Korean populations, while Byun et al. 9 assessed the mutation status of KRAS based on the location of primary tumors from 1,115 Korean patients. Therefore, there are further unmet needs to investigate the molecular characteristics along with accompanying clinical information for East Asian populations as well as for region or nation-specific populations. In this study, tumor samples from 172 Korean patients with colorectal cancer were examined with deep targeted sequencing to evaluate whether their various clinical characteristics are related with different molecular characteristics. We assessed the single-nucleotide variations from tumor samples to select potentially pathogenic somatic mutations. The mutational characteristics of the 172 patients were evaluated in association with multiple clinical features, including tumor locations, ages, cancer antigen marker levels, and the depth of tumor invasion. The identified mutational characteristics were also compared with that of the Caucasian colorectal cancer patients from independent data to show its ethnic specificity. The identified associations between somatic mutations and clinical features of Korean colorectal cancer patients from this study can provide beneficial information to better understand the molecular characteristics of colorectal cancer.

## Materials and Methods

### Patients and clinical data collection

FFPE samples of primary tumor tissues were obtained from 172 colorectal cancer patients and prepared for mutation profiling with deep targeted sequencing. The tumor tissue samples were retrieved from patients at Gachon University Gil Medical Center (Incheon, Republic of Korea) between April 2017 and April 2019. Patients were enrolled only when colorectal cancer was detected by abdominal and pelvic computed tomography to acquire enough specimens for this study. The detailed demographic and clinical characteristics of the 172 patients are given in Table 1. The experiments conducted on patient samples were approved by the institutional review board of Gachon University Gil Medical Center (IRB No. GCIRB2019-223, A study to identify the relationship between genetic variation of colon cancer and clinicopathological characteristics). All experiments and analysis procedures were performed in accordance with the relevant guidelines and regulations. Written informed consents were obtained from all participating patients.

Regarding the laboratory measurement of CA 19-9 and CEA levels, peripheral blood samples were obtained from participating patients at an average 8 ± 2 days prior to the operation. All the assays were performed in a single laboratory by following manufacturers' guidelines, where the RIAMAT SR-300 kit (Stratec, Germany) was used for the serum CEA levels, and the ADVIA Centaur CA 19-9 assay (Siemens Medical Solutions Diagnostics, US) was used to measure CA 19-9 levels. For the classification of MSI status, a panel of microsatellite markers, including the mononucleotide repeat markers BAT25 and BAT26, and the dinucleotide repeat markers D5S346, D2S123, and D17S250, were used according to the international guidelines. Instability in more than 40% of the analyzed markers was defined as MSI-high and classified as MSI in this study. Tumors with instability in less than 40% of the markers (MSI-low) and tumors without instability were classified as MSI 10.

### Molecular characterization with targeted sequencing

The molecular characterization of tumors was performed by identifying genomic mutations with targeted sequencing. Sequencing was performed on genomic DNA extracted from FFPE tumor samples using the NextSeq platform (Illumina, Inc., San Diego, CA), and no matched normal tissue was sequenced. The sequencing was performed at LabGenomics (Seongnam, Republic of Korea) using the CancerSCAN level 2 targeted panel. For sequencing, specific regions of the genome spanning 2.599 Mb were amplified to cover 375 whole-gene targets (Supplementary Table S1). The targeted regions were sequenced with average target-depth of 800X, and more than 94% of sequenced bases showed quality scores higher than Q30.

It is known that mutational profiling with FFPE samples can include false-positive mutations, where base changes of C to T or G to A can occur with low frequency due to cytosine deamination in the process of manufacturing FFPE samples 11. In order to exclude such potentially false-positive mutations due to the process of manufacturing FFPE samples, mutations of base changes C to T and G to A with variant allele fraction less than 5% were excluded from the downstream analysis 12.

### Filtering potential somatic mutations

In order to consider only potentially somatic mutations that affect protein formation and functions among the identified SNVs and short insertions/deletions, genetic variants whose positions are other than exons or splicing sites as well as synonymous variants were discarded from further analysis. Among the remaining variants, variants with minor allele frequencies greater than 0.01 from multiple variant databases (ESP6500, ExAC and 1000 genome project) were further discarded. As a final measure to further eliminate potentially false positive somatic mutations, the mutation frequencies of individual genes from the variants were computed from the 172 patients in this study, and they were compared with the mutation frequencies from the COSMIC database for colorectal cancer. If a gene showed more than 3-fold difference in mutation frequencies between our data and the COSMIC database or its statistical significance p-value was less than 0.05 (by Fisher's exact test), it was considered as a false-positive finding and discarded from the results.

### Statistical methods

Fisher's exact test was applied to evaluate the statistical significance of difference in mutation frequencies between two patient groups, and genes showing p-value < 0.05 were declared to show differences. TML of each sample was measured with the number of nonsynonymous mutations per 1 Mb sequenced bases, where expected hotspot mutations 13 were discarded before computation to avoid potential bias of enriched mutations from using the targeted sequencing panel. Welch's t-test was used to test if left- and right-sided tumors show differences in TML.

In order to evaluate if the identified mutational characteristics for each clinical variable from our study is specific to Korean populations, we compared our results with the mutational characteristics of the Caucasian population from the TCGA colorectal cancer data 14. Among the clinical variables that we investigated, information on tumor location, subject ages, BMI, TNM stages were also available from TCGA. Somatic mutation information for the 207 Caucasian subjects from the TCGA colorectal cancer data was used to identify the clinical variable-associated mutational characteristics. For each clinical variable, Caucasian subjects were grouped based on the same criteria with our Korean subjects, and Fisher's exact test was performed on mutation frequencies between subject groups. The same p-value threshold of 0.05 was used to declare statistical significance on the difference of mutation frequencies for the Caucasian subjects.

## Results

### Discrepancy in genomic characteristics of Korean colorectal cancers by tumor locations

**Figure 1:**
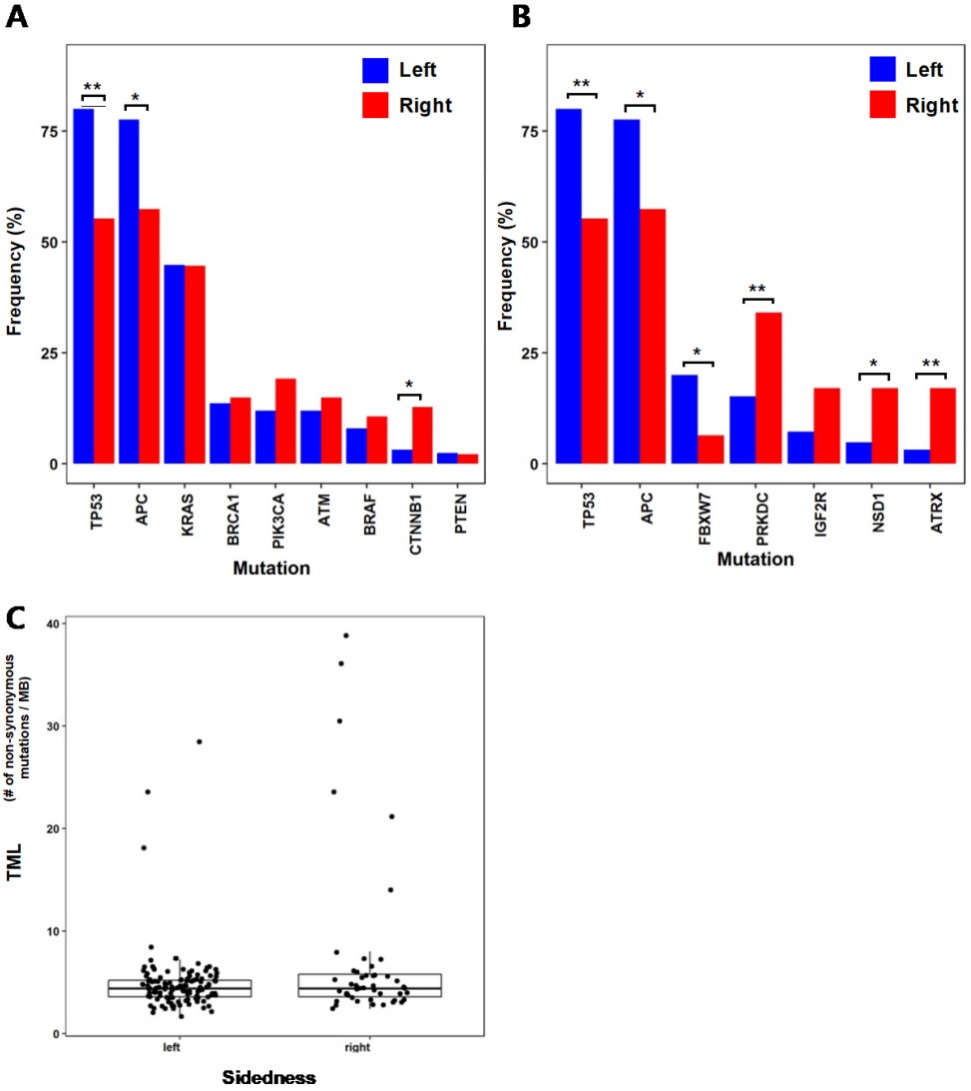
** Differences in mutational characteristics based on tumor locations** (* :p < 0.05, ** :p < 0.01) (A) Mutation frequencies of common colorectal cancer genes for the left-sided and right-sided tumors (B) Mutation frequencies of selected genes with large frequency differences (C) Tumor mutational load (TML) of the left-sided and the right-sided tumors

We compared the mutation frequencies between 125 patients with left-sided tumors and 47 patients with right-sided tumors. When comparing the mutation frequencies of selected genes (BRAF, KRAS, TP53, APC, PIK3CA, CTNNB1, ATM, PTEN and BRCA1) that are commonly known for colorectal cancer 15 (Fig. 1A), TP53 (left = 80%, right = 55.3%; p = 0.002) and APC (left = 77.6%, right = 57.5%; p = 0.013) showed significant difference between left-sided and right-sided tumors with higher mutation frequencies on the left-sided tumors. On the contrary, CTNNB1 (left = 3.2%, right = 12.8%; p = 0.026) showed higher mutation frequency on the right-sided tumors than the left-sided tumors. Other study also investigated the differences in mutation frequencies of colorectal cancer based on tumor location 15. In that study, TP53 also showed significantly higher mutation frequency on the left-sided tumors than the right-sided tumors, which is consistent with our result. Their results also showed the same left/right-side preference of mutation frequencies for APC and CTNNB1, but their result did not accompany statistical significance. This slight difference between two studies may be due to the heterogeneity in genomic characteristics between subjects from Korean populations of our study and the subjects from the previous study. We also investigated other genes that showed large differences in mutation frequencies between the left-sided and right-sided tumors (Fig. 1B). FBXW7 (left = 20%, right = 6.4%; p = 0.036), which is known as a tumor suppressor gene, showed a significantly higher mutation frequency from the left-sided tumors than the right-sided tumors. PRKDC, ATRX, and NSD1 showed higher mutation frequencies from the right-sided tumors than the left-sided tumors. PRKDC (left = 15.2%, right = 34%; p = 0.01) is a protein kinase and plays a role in DNA double strand break repair 16. ATRX (left = 3.2%, right = 17%; p = 0.004) mutations have been reported in various types of cancer, and it is known to be associated with MSI 17. These higher mutation frequencies of PRKDC and ATRX from the right-sided tumors than the left-sided may be related to the more frequent observation of MSI-high cases from the colorectal cancer patients with right-sided tumors. NSD1 (left = 4.8%, right = 17%; p = 0.023) is known to play a role in histone methylation and its inactivation has been reported to lead to multiple cancers 18.

TML of each sample was calculated, and its distribution was compared between the left-sided and right-sided tumors (Fig. 1C). Difference between the two TML distributions does not show statistical significance (p = 0.059 by Welch's T-test), but the right-sided tumors including samples of very high mutational burdens show higher mutational burdens than the left-sided tumors on average (TML of right-sided tumors = 7.41 nonsynonymous mutations/megabase, TML of left-sided tumors = 4.98 nonsynonymous mutations/megabase). This may be also related to frequent MSI-High observations from right-sided colorectal cancers.

### Frequent mutations in early onset colorectal cancer

**Figure 2:**
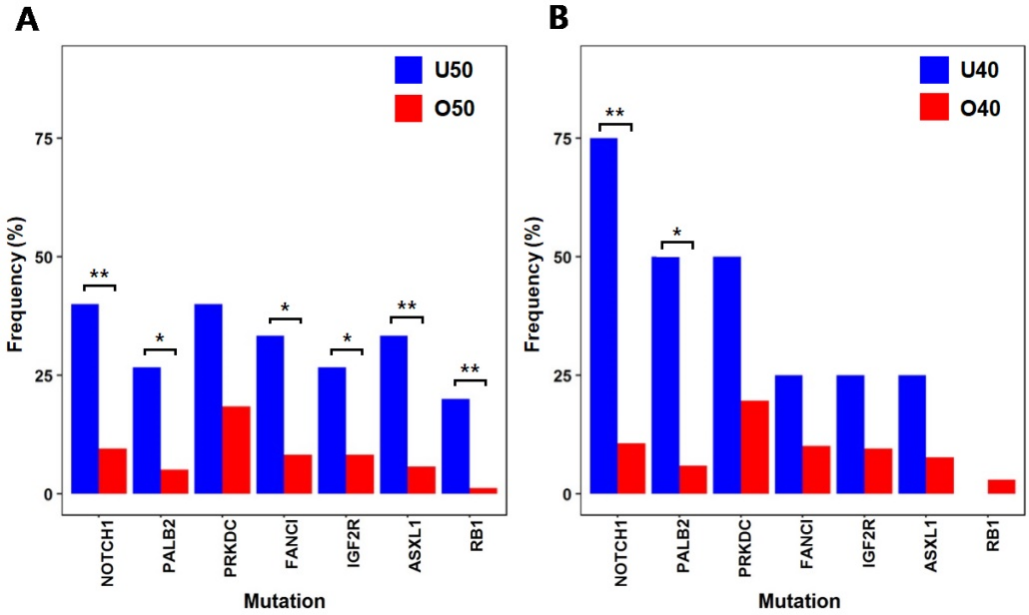
** Selected genes that show large differences in mutation frequencies based on patients' ages** (* :p < 0.05, ** :p < 0.01) (A) Mutation frequencies from two patient groups classified by age 50 (B) Mutation frequencies from two patient groups classified by age 40

To identify mutations associated with early-onset colorectal cancer, we compared the mutation frequencies of 15 patients with ages under 50 (U50) and 157 patients with ages 50 or older (O50) (Fig. 2A). NOTCH1(U50 = 40%, O50 = 9.6%; p = 0.004), RB1(U50 = 20%, O50 = 1.3%; p = 0.045), ASXL1(U50 = 33.4%, O50 = 5.7%; p = 0.003) and IGF2R(U50 = 26.7%, O50 = 8.3%; p = 0.045), which have been reported to be associated with various cancers as tumor suppressors 19-22, showed high frequencies of mutations in younger patients (U50). FANCI (U50 = 33.4%, O50 = 8.3%; p = 0.011) and PALB2 (U50 = 26.7%, O50 = 5.1%; p = 0.012), where they are known to play roles in repairing DNA double strand breaks 23, 24, also showed high frequencies of mutations from the younger U50 group. With this observation of higher mutation frequencies of some tumor suppressors and DNA repair genes, we further assessed the frequencies of mutations with more stringent categorization with a lower threshold of age 40 (four patients with ages under 40 (U40) VS. 168 patients with ages of 40 or older (O40)) (Fig. 2B). From the comparison, there were significant differences in mutation frequencies of NOTCH1 (U40 = 75%, O40 = 10.7%; p = 0.006) and PALB2 (U40 = 50%, O40 = 6%; p = 0.024). As these tumor suppressor and repairer of DNA strand break show higher mutation frequencies in younger patients, they may be involved in early initiation and development of colorectal cancer by losing tumor suppressing function and allowing more DNA structure alterations.

### Mutations in association with cancer antigen markers

**Figure 3:**
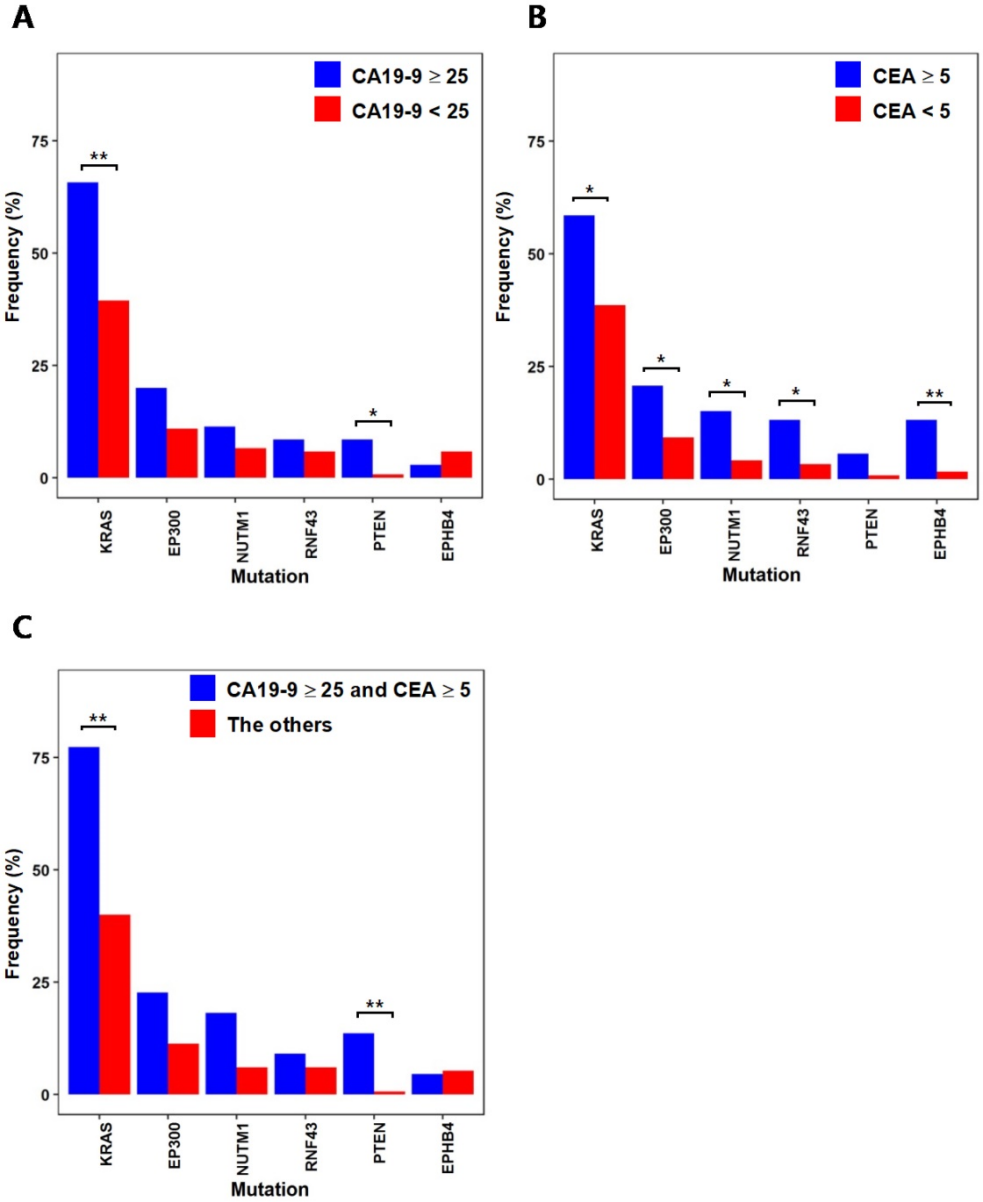
** Differences in mutational characteristics based on cancer antigen markers from six selected genes that showed significant difference** (* :p < 0.05, ** :p < 0.01) (A) Mutation frequencies from patient groups categorized by CA19-9 levels (B) Mutation frequencies from patient groups categorized by CEA levels (C) Mutation frequencies from the patient group with advanced colorectal cancer (CA19-9 ≥ 25 and CEA ≥ 5) and the other patients

CA19-9 and CEA are used as biomarkers for the diagnosis and prognosis of colorectal cancer. When comparing the mutation frequencies of individual genes from the 35 patients with CA19-9 levels greater than or equal to 25 with the mutation frequencies from the other 137 patients with CA19-9 levels lower than 25 (Fig. 3A), KRAS(CA19-9 level ≥ 25: 65.7%, CA19-9 level < 25: 39.4%; p = 0.007) and PTEN(CA19-9 level ≥ 25: 8.6%, CA19-9 level < 25: 0.7%, p = 0.03) showed significantly higher mutation frequencies. It is noteworthy that these two genes are known as common colorectal cancer mutations 15 and show correlated mutation frequencies with high CA19-9 levels. We also compared the mutation frequencies from the 53 patients with CEA levels greater than or equal to 5 with the mutation frequencies from the other 119 patients with CEA levels lower than 5 (Fig. 3B). KRAS (CEA level ≥ 5: 58.5%, CEA level < 5: 38.7%; p = 0.02) showed significantly high mutation frequency from the patients with the CEA level ≥ 5, while PTEN (CEA level ≥ 5: 5.7%, CEA level < 5: 0.8%; p = 0.087) did not show significant difference. Besides KRAS, we also observed that several cancer-associated genes show more frequent mutations from the patients with the CEA level ≥ 5. RNF43 (CEA level ≥ 5: 13.2%, CEA level < 5: 3.4%; p = 0.036) is known as a tumor suppressor and negatively regulates WNT signaling 25. EP300 (CEA level ≥ 5: 20.8%, CEA level < 5: 9.2%; p = 0.048) is a histone acetyltransferase, where its role in cell proliferation and differentiation can be associated with cancer 26. EPHB4(CEA level ≥ 5: 13.2%, CEA level < 5: 1.7%, p = 0.004) mutation was also more frequent from the patients with the CEA level ≥ 5, where previous studies have shown that it acts as a tumor suppressor 27. By simultaneously considering both of CA19-9 and CEA levels, we evaluated the mutation frequencies from the 22 patients with high CA19-9 (≥ 25) and CEA (≥ 5) levels in comparison with the other 150 patients to characterize the mutations from patients with more advanced colorectal cancer. From the comparison, KRAS (CA19-9 ≥ 25 and CEA ≥ 5: 77.3%, the others: 40%; p = 0.001) and PTEN (CA19-9 ≥ 25 and CEA ≥ 5: 13.7%, the others: 0.7%; p = 0.007) showed significantly higher mutation frequencies from the 22 patients with advanced colorectal cancer. It should be noted that the mutation frequencies of KRAS and PTEN were more significantly higher from patients with both antigen marker levels above thresholds than the patients categorized with only one antigen marker levels (see Fig. 3C in comparison with Figs. 3A and 3B).

### Mutational characteristics by the depth of tumor invasion

**Figure 4:**
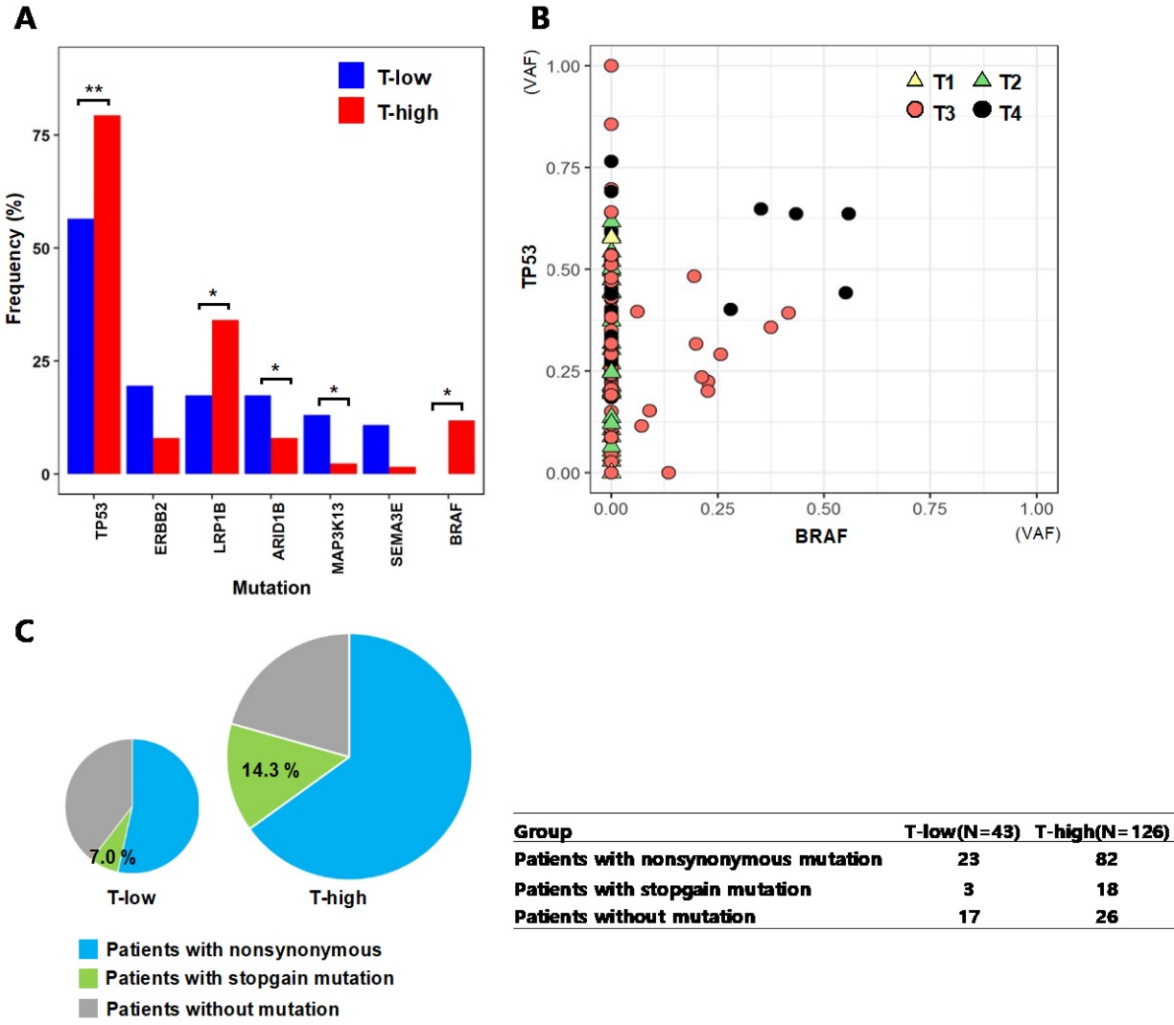
**Mutational characteristics by tumor stages from the top seven genes that showed large differences in mutation frequencies** (A) Mutation frequencies from patient groups categorized by T stages (T-low: T1 and T2, T-high: T3 and T4) (* :p < 0.05, ** :p < 0.01) (B) Variant allele frequencies (VAFs) of TP53 and BRAF by T stages (C) The fractions of nonsynonymous and stopgain mutations of TP53 from the T-low and T-high patient groups. The size of the pie chart represents the number of patients in each group.

The T stage of tumor represents the depth of invasion into nearby tissues, where a higher T stage indicates a deeper invasion of the tumor. We categorized the patients into two groups of 46 patients with low T stages (T-low; T1 and T2) and 126 patients with high T stages (T-high; T3 and T4), and compared the mutation frequencies of individual genes between the two groups (Fig. 4A). TP53(T-low: 56.5%, T-high: 79.4%; p = 0.006) and BRAF (T-low: 0%, T-high: 11.9%; p = 0.012), which are known frequently mutated genes in colorectal cancer, showed more frequent mutations in the patient group of T3 and T4 stages (T-high). LRP1B mutation (T-low: 17.4%, T-high: 34.1%; p = 0.038) was also more frequent at T3 and T4 stages, and this gene has been reported to inhibit the progression of colorectal cancer cells 28. For TP53 and BRAF, their VAFs were assessed based on the T stages of patients' tumors (Fig. 4B). The mutation of TP53 resides across all T stages from T1 to T4, while it is notable that the BRAF mutations were observed in only advanced tumors of T stages T3 and T4. For the tumors with both of TP53 and BRAF mutations, the VAFs of these two genes are generally higher in T4 tumors than the VAFs in T3 tumors, thus implying that more advanced tumors have higher fraction of cancer cells with TP53 and BRAF mutations. We also assessed the fractions of TP53 mutation types from T-low and T-high patient groups (Fig. 4C). The patients in the T-high group had TP53 stopgain mutations with a fraction (14.3%) more than twice of patients from the T-low group (7.0%), even though the difference was not statistically significant (p = 0.199).

### Genetic mutations correlated with other clinical variables

We investigated mutations correlated with several additional clinical variables, where the mutation frequencies of individual genes were compared between patient groups that were categorized by each clinical variable (Table 2). As metastasis is one of major factors of poor prognosis for patients with colorectal cancer, mutation frequencies were compared between the group of patients without metastasis (M0, N = 144) and the other group of patients with metastasis (M1, N = 28). BRAF mutations (M0: 6.3%, M1: 21.4%; p = 0.019) are more frequent in patients with metastasis, and this is a higher frequency compared to previously reported 5-10% in metastatic colorectal cancer 29. EPHA6 (M0: 4.9%, M1: 17.9%; p = 0.028) is an ephrin receptor and has a high frequency of mutations in the group of patients with metastasis, and its association with metastasis has been previously reported for colorectal cancer 30. We also compared the mutation frequencies the group of patients without regional lymph node metastasis (N0, N = 93) and the other group of patients with regional lymph node metastasis (N1, N = 79). The well-known APC gene for colorectal cancer 30 showed a higher mutation frequency (N0: 65.6%, N1: 79.8%; p = 0.042) in patients with regional lymph node metastasis. ETV4(N0: 0%, N1: 8.9%; p = 0.004) also showed higher mutation frequency from patients with regional lymph node metastasis. Microsatellite instability is an important genetic marker in colorectal cancer, and it is known that patients with microsatellite instability have a better prognosis than those who do not. We compared the mutation frequencies from the microsatellite instability patient group (MSI, N = 7) with the frequencies from the microsatellite stability patient group (MSS, N = 165). TP53 (MSI: 28.6%, MSS: 75.2%; p = 0.015) and APC (MSI: 28.6%, MSS: 73.9%; p = 0.019) are known to be commonly mutated in colorectal cancer 15, and they showed higher mutation frequencies from the MSS group. Considering that patients with MSS generally have worse prognosis than patient of MSI, the more frequent mutations of these tumor suppressor genes from the MSS group may imply more aggressive tumorigenesis. LVI is considered as a prognostic factor and was reported to affect the survival rate of patients with colorectal cancer. From comparing the mutation frequencies in the 91 patients with LVI to the frequencies in the 81 patients without LVI, TP53 mutation (with LVI: 82.4%, without LVI: 63%; p = 0.006) was more frequent in the group of patients with LVI. This is another evidence that the mutation of the tumor suppressor TP53 is correlated with poor prognosis of colorectal cancer. Insulin receptor INSR (with LVI: 9.9%, without LVI: 1.2%; p = 0.02) also showed high mutation frequency from the patients with LVI. For correlating mutation frequencies from these colorectal cancer patients with the accompanying diagnosis status of diabetes, mutation frequencies of individual genes from 38 patients with type 2 diabetes (DM) were compared with the mutation frequencies from the other 134 patients without type 2 diabetes (non-DM). ARID1B is a chromatin remodeling factor and known to inhibit WNT/β-catenin signaling 31, and it showed more frequent mutations in the diabetic group (DM: 21.1%, non-DM: 7.5%; p = 0.03). TNKS2 (DM: 18.4%, non-DM: 1.5%; p = 0.0004) also showed a high frequency of mutations in the diabetic group, and it is known to be associated with the incidence of small cell lung cancer 32. TNKS2 is known to play a role in the translocation of GLUT4, a type of glucose transporter 33, and this suggests that there can be certain impairment of glucose transport from patients with TNKS2 mutations. Regarding the status of obesity, we compared the mutation frequencies of individual genes from 45 patients with BMI ≥ 25 to the mutation frequencies from the other 127 patients with BMI < 25. RAD50 (BMI ≥ 25: 15.6%, BMI < 25: 4.7%; p = 0.042), a double strand break repair gene, showed a significantly higher mutation frequency in the obese patient group. This gene is known to be associated with breast cancer 34. IRS2 (BMI ≥ 25: 8.8%, BMI < 25: 1.5%; p = 0.041) is a gene involved in PI3K signaling that is related to colorectal cancer 14, and it is also known to be at the center of pathophysiology in diabetes where the aberration of IRS may be a fundamental cause of the development of insulin resistance, obesity, β cell failure, and type 2 diabetes 35.

### Ethnic specificity of Korean mutational characteristics in comparison with the Caucasian mutational characteristics

For each clinical information that is commonly available between our Korean subjects and the TCGA Caucasian subjects, the number of mutation genes that are significantly associated with the clinical variable is listed in Table 3 for each ethnic group along with the number of common genes between the Korean and Caucasian subjects. The complete lists of genes that showed different mutation frequencies based on clinical information criteria are given in Supplementary materials for the Korean subjects (Supplementary Table S2) and the TCGA Caucasian subjects (Supplementary Table S3).

For the mutation genes that showed significantly different mutation frequencies by tumor locations, only five genes were common between the Korean and the Caucasian. APC is a well-known gene that are often mutated in colorectal cancer, and the mutation frequency was significantly higher in left-sided tumors for both of Korean (left: 77.6%, right: 57.4%, p = 0.013) and Caucasian (left: 84.3%, right: 60.8%, p=0.001). JAK 1 is known as a gene involved in cancer-related JAK-STAT pathway signaling, and it showed higher mutation frequencies in right-sided tumors for both of Korean (left: 1.6%, right: 8.5%, p = 0.048) and Caucasian (left: 0%, right: 10.3%; p=0.005). For the regional lymph node metastasis status (presence - N1, absence - N0), ACVR1B and PIK3CG showed similarly low mutation frequencies in tumors with regional lymph node metastasis for both of Korean (ACVR1B - N1: 1.3%, N0: 9.7%, p = 0.022; PIK3CG - N1: 0%, N0: 7.5%, p = 0.016) and Caucasian (ACVR1B - N1: 1.1%, N0: 8.6%, p = 0.025; PIK3CG - N1: 2.2%, N0: 12.1%, p = 0.008). For age, BMI, TNM T and M stages, there was no common mutation gene of clinical variable association between the two ethnic groups.

## Discussion

Our study shows that using deep targeted sequencing for the molecular characterization of colorectal cancer can reveal various mutational patterns that can be correlated with clinical features. Although numerous genomic studies have been reported for colorectal cancer, few studies explored the link between mutational characteristics and various clinical features from Korean populations using next generation sequencing. From our study, TP53 and APC showed significantly higher mutation frequencies on the left-sided tumors than the right-sided tumors. A previous report 15 also showed significantly higher mutation frequency of TP53 from the left-sided tumors, but they did not report the preferential mutation of APC on the left-sided tumors with statistical significance. This discrepancy from our study in comparison with the previous report can be due to the heterogeneity in genomic characteristics from Korean populations. On the other hand, PRKDC and ATRX genes showed significantly higher mutation frequencies from the right-sided tumors. Both of these genes can be associated with more frequent observation of highly mutated MSI-high cases with right-sided tumors, as PRKDC plays a role in DNA double strand break repair 1 and ATRX is known to be associated with MSI 36. This large molecular difference between the right and left-sided colorectal tumors may be due to different embryonic origins of left and right colon. Early onset colorectal cancer patients from our study showed more frequently mutated NOTCH1 tumor suppressor and the DNA strand break repair gene PALB2, and mutations of these genes can be related to the early tumor initiation and generally worse prognosis from young colorectal cancer patients. When the patients were categorized with the cancer antigen markers, patients with advanced colorectal cancer had more frequent KRAS and PTEN mutations. The mutation of KRAS is related with the resistance to EGFR-targeted therapeutics, and the loss of the tumor suppressor PTEN implies more aggressive tumorigenesis from these patients with advanced colorectal cancer. Another category of tumor development is the T stage that represents the depth of tumor invasion, and tumors with deeper invasion (T3 and T4) showed more frequent mutations of TP53 and BRAF. While the mutations of TP53 were observed across all T stages, only advanced tumors of T3 and T4 stages had BRAF mutations. Moreover, the VAFs of TP53 and BRAF were higher from the T4 tumors than the T3 tumors, and this suggests that advanced tumors with deeper invasion have more fraction of cancer cells with the mutations of these two genes. Regarding TP53, the ratio of patients with stopgain mutations were higher from T3 and T4 stages than from earlier T stages, and this implies the correlation between the loss of TP53 tumor suppressor and more aggressive tumor progression of colorectal cancer. In order to investigate the ethnic specificity of the identified mutational characteristics of the Korean subjects, we also analyzed the mutational characteristics of clinical association from the Caucasian subject samples of the TCGA study. The comparison of clinical variable-associated mutation genes between the two ethnic groups showed little overlap (Table 3), and only a few key colorectal cancer genes such as APC and JAK1 were common. This suggests that our results imply mutational characteristics of colorectal cancer that are specific to the Korean population in general.

We assessed the mutational characteristics of colorectal cancer from Korean patients, but there are certain limitations in our study. Firstly, we did not characterize structural variations including CNVs, as only limited successes have been reported in identifying structural variations using targeted sequencings. Secondly, our study did not incorporate matched normal DNA and this limits the ability to detect more accurate somatic mutations from tumors. We applied stringent criteria to filter out potential germline mutations that could have been included as false-positive mutations. However, there is no method to accurately identify somatic mutations without normal DNA, and our result may include certain level of variances. Even with these limitations, we consider that our study can provide beneficial information on mutational characteristics from Korean colorectal cancer patients, and it will improve our understanding on the clinical and molecular aspects of colorectal cancer.

## Supplementary Material

Supplementary tables.

## Figures and Tables

**Table 1:** Demographic and clinical landscape of the 172 CRC patients in this study

	Characteristics	Number of patients (%)
Sex	Male	112 (65.1%)
	Female	60 (34.9%)
	
Tumor sidedness	Left	125 (72.7%)
	Right	47 (27.3%)
	
Age	>=50	157 (91.3%)
	<50, >=45	11 (6.4%)
	>45	4 (2.3%)
	
CA19-9	>=25	35 (20.3%)
	<25	137 (79.7%)
	
CEA	>=5	53 (30.8%)
	<5	119 (69.2%)
	
TNM T Stage	T1	19 (11.0%)
	T2	27 (15.7%)
	T3	94 (54.7%)
	T4	32 (18.6%)
	
TNM N Stage	N0	93 (54.1%)
	N1	79 (45.9%)
	
TNM M stage	M0	144 (83.7%)
	M1	28 (16.3%)
	
MSI	MSS	165 (95.9%)
	MSI	7 (4.1%)
	
Lymphovascular invasion (LVI)	Without LVI	81 (47.1%)
	With LVI	91 (52.9%)
	
Diabetes mellitus	Without diabetes mellitus	134 (77.9%)
	With diabetes mellitus	38 (22.1%)
	
BMI	>=25	45 (26.2%)
	<25	127 (73.8%)
	
ECOG performance status	0	51 (29.7%)
	1	97 (56.4%)
	2	24 (13.9%)
	
Synchronous lung metastasis	No	169 (98.3%)
	Yes	3 (1.7%)
	
Synchronous liver metastasis	No	155 (90.1%)
	Yes	17 (9.9%)
	
Bowel obstruction	No	128 (74.4%)
	Yes	44 (25.6%)
	

**Table 2:** List of significant mutations that showed different frequencies between patient groups by selected clinical characteristics

Genes	Categorization by clinical variable		p-value
	Without metastasis	With metastasis	
	(N=144)	(N=28)	
BRAF	9, 6.3%	6, 21.4%	0.019
EPHA6	7, 4.9%	5, 17.9%	0.028
	Without regional lymph node metastasis	With regional lymph node metastasis	
	(N=93)	(N=79)	
APC	61, 65.6%	63, 79.8%	0.042
ETV4	0, 0%	7, 8.9%	0.004
	Microsatellite stable	Microsatellite instability	
	(N=165)	(N=7)	
TP53	124, 75.2%	2, 28.6%	0.015
APC	122, 73.9%	2, 28.6%	0.019
	Without lymphovascular invasion	With lymphovascular invasion	
	(N=81)	(N=91)	
TP53	51, 63.0%	75, 82.4%	0.05
INSR	1, 1.2%	9, 9.9%	0.02
	Without diabetes mellitus	with diabetes mellitus	
	(N=134)	(N=38)	
ARID1B	10, 7.5%	8, 21.1%	0.031
TNKS2	2, 1.5%	7, 18.4%	0.0004
	BMI ≥ 25	BMI < 25	
	(N=45)	(N=127)	
RAD50	7, 15.6%	6, 4.7%	0.042
IRS2	4, 8.8%	2, 1.5%	0.041

**Table 3:** Number of genes that showed significantly different mutation frequencies by each clinical information criteria

Clinical information	Associated mutation genes in Korean	Associated mutation genes in Caucasian	Associated mutation genes in both ethnic groups
Tumor location (Left-sided VS. Right-sided)	22	59	5
Age (ages under 50 VS. ages 50 or older)	11	8	0
BMI (BMI ≥ 25 VS. BMI < 25)	6	15	0
TNM T stage (Low T stage(T1, T2) VS. High T stage(T3, T4))	9	2	0
TNS N stage (Without regional lymph node metastasis VS. With regional lymph node metastasis)	11	30	2
TNM M stage (Without metastasis VS. With metastasis)	5	0	0

## Abbreviations

VS.: versus
CMS: consensus molecular subtype
MSI: microsatellite instability
FFPE: formalin-fixed paraffin-embedded
IRB: institutional review board
Mb: mega bases
TML: tumor mutational load
CA: carbohydrate antigen
CEA: carcinoembryonic antigen
VAF: variant allele frequency
MSS: microsatellite stability
LVI: lymphovascular invasion
DM: type 2 diabetes mellitus
BMI: body mass index
CNV: copy number variation
