# Supplementary material for: Mutational Characterization of Colorectal Cancer from Korean Patients with Targeted Sequencing
Source: J Cancer. 2021 Oct 28;12(24):7300–10. doi: 10.7150/jca.61324 (PMC8734410; doi:10.7150/jca.61324)
Supplement: Supplementary file 1 — Supplementary tables. [file jcav12p7300s1.pdf]

**Article title**

# Mutational characterization of colorectal cancer from Korean patients with targeted sequencing

**Journal name**

Journal of Cancer

**Author information**

Jongmin Lee, MS[1,2*], Sangtae Choi, MD, PhD[3*], Donghae Jung, MD, PhD[4], YunJae Jung, MD, PhD[2,5], Jung Ho Kim, MD, PhD[6], Sungwon Jung, PhD[1,2,7c], and Won-Suk Lee, MD, PhD[3c]

[1]Gachon Institute of Genome Medicine and Science, Gachon University Gil Medical Center, Incheon, Republic of Korea

[2]Gachon Advanced Institute for Health Science and Technology, Gachon University, Incheon, Republic of Korea

[3]Department of Surgery, Gachon University College of Medicine Gil Medical Center, Incheon, Republic of Korea

[4]Department of Pathology, Gachon University College of Medicine Gil Medical Center, Incheon, Republic of Korea

[5]Department of Microbiology, Gachon University College of Medicine, Incheon, Republic of Korea

[6]Department of Internal Medicine, Gachon University College of Medicine Gil Medical Center, Incheon, Republic of Korea

[7]Department of Genome Medicine and Science, Gachon University College of Medicine, Incheon, Republic of Korea

*: These authors equally contributed as first authors.

c: Corresponding Authors: Sungwon Jung (e-mail: sjung@gachon.ac.kr; ORCID: 0000-0001-6002-554X) and Won-Suk Lee (e-mail: lws@gilhospital.com)

**Supplementary Table S1.** List of genes covered by the targeted sequencing

| |
|---|
| ABL1, ABL2, ACVR1B, ADGRA2, AKT1, AKT2, AKT3, ALK, ALOX12B, AMER1, APC, APCDD1, APOBEC3A, APOBEC3B, AR, ARAF, ARFRP1, ARID1A, ARID1B, ARID2, ASXL1, ATM, ATR, ATRX, AURKA, AURKB, AXIN1, AXL, B2M, BACH1, BAP1, BARD1, BCL2, BCL2A1, BCL2L1, BCL2L2, BCL6, BCOR, BCORL1, BCR, BLM, BRAF, BRCA1, BRCA2, BRD2, BRD3, BRD4, BRIP1, BTG1, BTK, CARD11, CASP8, CBFB, CBL, CCND1, CCND2, CCND3, CCNE1, CD79A, CD79B, CDC42, CDC73, CDH1, CDH2, CDH20, CDH5, CDK12, CDK4, CDK6, CDK8, CDKN2A, CDKN2B, CDKN2C, CDX2, CEBPA, CHD1, CHD2, CHD4, CHEK1, CHEK2, CHUK, CIC, CRBN, CREBBP, CRKL, CRLF2, CSF1R, CTCF, CTNNA1, CTNNB1, CUL4A, CUL4B, CYLD, CYP17A1, DAXX, DDR2, DIS3, DNMT1, DNMT3A, DOCK2, DOT1L, EGFR, ELMO1, EMSY, EP300, EPHA3, EPHA5, EPHA6, EPHA7, EPHB1, EPHB4, EPHB6, ERBB2, ERBB3, ERBB4, ERCC2, ERG, ESR1, ETV1, ETV4, ETV5, ETV6, EWSR1, EYA2, EZH2, FAM46C, FANCA, FANCC, FANCD2, FANCE, FANCF, FANCG, FANCI, FANCL, FANCM, FAT3, FBXW7, FGF10, FGF12, FGF14, FGF19, FGF23, FGF3, FGF4, FGF6, FGF7, FGFR1, FGFR2, FGFR3, FGFR4, FLT1, FLT3, FLT4, FOXA1, FOXL2, FUBP1, GATA1, GATA2, GATA3, GID4, GNA11, GNA13, GNAQ, GNAS, GRIN2A, GSK3B, GUCY1A2, H3F3A, HGF, HIST1H3B, HNF1A, HOXA3, HRAS, HSP90AA1, IDH1, IDH2, IGF1, IGF1R, IGF2R, IKBKE, IKZF1, IL7R, INHBA, INPP4B, INSR, IRF4, IRS2, ITK, JAK1, JAK2, JAK3, JUN, KAT6A, KDM5A, KDM5C, KDM6A, KDR, KEAP1, KIT, KLF4, KLHL6, KRAS, LMO1, LRP1B, LRP6, LTK, MAML1, MAP2K1, MAP2K2, MAP2K4, MAP3K1, MAP3K13, MCL1, MDM2, MDM4, MED12, MEF2B, MEN1, MET, MITF, MLH1, MPL, MRE11A, MSH2, MSH6, MTOR, MUTYH, MYC, MYCL, MYCN, MYD88, NCOA3, NCOR1, NF1, NF2, NFE2L2, NKFBIA, NKX2-1, NKX3-1, NOTCH1, |

NOTCH2, NOTCH3, NOTCH4, NPM1, NRAS, NSD1, NTRK1, NTRK2, NTRK3, NUP93, NUTM1, PAK3, PAK7, PALB2, PARP1, PARP2, PARP3, PARP4, PAX5, PBRM1, PDGFRA, PDGFRB, PDK1, PGR, PHF6, PHLPP2, PIK3C3, PIK3CA, PIK3CG, PIK3R1, PIK3R2, PKHD1, PLCG1, PMS2, PNRC1, PPP2R1A, PRDM1, PRKAR1A, PRKDC, PRPF40B, PRSS8, PTCH1, PTCH2, PTEN, PTPN11, PTPRD, RAB35, RAC1, RAC2, RAD50, RAD51, RAD51B, RAD51C, RAD51D, RAD52, RAD54L, RAF1, RARA, RB1, REL, RET, RICTOR, RNF43, ROBO1, ROBO2, ROS1, RPA1, RPTOR, RUNX1, RUNX1T1, SEMA3A, SEMA3E, SETBP1, SETD2, SF3A1, SF3B1, SH2B3, SKP2, SLIT2, SMAD2, SMAD3, SMAD4, SMARCA1, SMARCA4, SMARCB1, SMARCD1, SMO, SOCS1, SOX10, SOX2, SOX9, SPEN, SPOP, SRC, SRSF1, SRSF2, SRSF7, STAG2, STAT3, STAT4, STK11, SUFU, SYK, TBX22, TBX3, TERT, TET2, TGFBR2, TIPARP, TMPRSS2, TNFAIP3, TNFRSF14, TNKS, TNKS2, TOP1, TP53, TRAF7, TRRAP, TSC1, TSC2, TSHR, U2AF1, U2AF2, USP9X, VHL, WHSC1L1, WISP3, WT1, WWP1, XBP1, XPO1, XRCC3, ZNF217, ZNF703, ZRSR2

**Supplementary Table S2.** Genes that show significantly different mutation frequencies by each clinical information criteria from tumors of the 172 Korean patients with colorectal cancer. Genes in boldface presentation are the common genes that also showed significantly different mutation frequencies from the Caucasian patients of the TCGA colorectal cancer data set.

| Genes | Categorization by clinical variable | | p-value |
|---|---|---|---|
| | Left-sided (N=125) | Right-sided (N=47) | |
| **APC** | **97, 77.6%** | **27, 57.4%** | **0.013** |
| **APCDD1** | **0, 0.0%** | **3, 6.4%** | **0.019** |
| ATRX | 4, 3.2% | 8, 17.0% | 0.004 |
| CASP5 | 0, 0.0% | 3, 6.4% | 0.019 |
| CTNNB1 | 4, 3.2% | 6, 12.8% | 0.026 |
| DDX27 | 0, 0.0% | 3, 6.4% | 0.019 |
| FBXW7 | 25, 20.0% | 3, 6.4% | 0.036 |
| **HNF1A** | **2, 1.6%** | **4, 8.5%** | **0.048** |
| **INSR** | **4, 3.2%** | **6, 12.8%** | **0.026** |
| **JAK1** | **2, 1.6%** | **4, 8.5%** | **0.048** |
| MCHR2 | 0, 0.0% | 3, 6.4% | 0.019 |
| NSD1 | 6, 4.8% | 8, 17.0% | 0.023 |
| NSD3 | 1, 0.8% | 4, 8.5% | 0.020 |
| PIK3C3 | 0, 0.0% | 3, 6.4% | 0.019 |
| PRKDC | 19, 15.2% | 16, 34.0% | 0.010 |
| RAF1 | 0, 0.0% | 3, 6.4% | 0.019 |

| | | | |
|---|---|---|---|
| RB1 | 1, 0.8% | 4, 8.5% | 0.020 |
| RPA1 | 4, 3.2% | 6, 12.8% | 0.026 |
| SLC16A4 | 0, 0.0% | 3, 6.4% | 0.019 |
| TMEM97 | 0, 0.0% | 3, 6.4% | 0.019 |
| TP53 | 100, 80.0% | 26, 55.3% | 0.002 |
| TTC3 | 0, 0.0% | 3, 6.4% | 0.019 |

| | ages under 50 (N=157) | ages 50 or older (N=15) | |
|---|---|---|---|
| ALOX12B | 3, 1.9% | 3, 20.0% | 0.009 |
| ASXL1 | 9, 5.7% | 5, 33.3% | 0.003 |
| FANCE | 0, 0.0% | 2, 13.3% | 0.007 |
| FANCI | 13, 8.3% | 5, 33.3% | 0.011 |
| GID4 | 2, 1.3% | 2, 13.3% | 0.039 |
| IGF2R | 13, 8.3% | 4, 26.7% | 0.045 |
| ITK | 2, 1.3% | 2, 13.3% | 0.039 |
| NOTCH1 | 15, 9.6% | 6, 40.0% | 0.004 |
| PALB2 | 8, 5.1% | 4, 26.7% | 0.012 |
| PIK3R2 | 2, 1.3% | 2, 13.3% | 0.039 |
| RB1 | 2, 1.3% | 3, 20.0% | 0.005 |

| | CA19-9 level ≥ 25 (N=35) | CA19-9 level < 25 (N=137) | |
|---|---|---|---|
| ARID1B | 0, 0.0% | 18, 13.1% | 0.026 |
| CHEK1 | 3, 8.6% | 0, 0.0% | 0.008 |
| ETV5 | 3, 8.6% | 1, 0.7% | 0.027 |

| | | | |
|---|---|---|---|
| KRAS | 23, 65.7% | 54, 39.4% | 0.007 |
| PRKDC | 2, 5.7% | 33, 24.1% | 0.017 |
| PTEN | 3, 8.6% | 1, 0.7% | 0.027 |
| | CEA level ≥ 5 (N=53) | CEA level < 5 (N=119) | |
| CASP5 | 3, 5.7% | 0, 0.0% | 0.028 |
| EP300 | 11, 20.8% | 11, 9.2% | 0.048 |
| EPHB4 | 7, 13.2% | 2, 1.7% | 0.004 |
| KDM6A | 5, 9.4% | 2, 1.7% | 0.029 |
| KRAS | 31, 58.5% | 46, 38.7% | 0.020 |
| MSH3 | 4, 7.6% | 1, 0.8% | 0.032 |
| NUTM1 | 8, 15.1% | 5, 4.2% | 0.024 |
| RAD51D | 3, 5.7% | 0, 0.0% | 0.028 |
| RNF43 | 7, 13.2% | 4, 3.4% | 0.036 |
| SMAD3 | 5, 9.4% | 0, 0.0% | 0.002 |
| SMARCA1 | 4, 7.6% | 1, 0.8% | 0.032 |
| SOX2 | 3, 5.7% | 0, 0.0% | 0.028 |
| | Low T stage(T1, T2) (N=46) | High T stage(T3, T4) (N=126) | |
| ALOX12B | 4, 8.7% | 2, 1.6% | 0.045 |
| BRAF | 0, 0.0% | 15, 11.9% | 0.012 |
| INHBA | 4, 8.7% | 2, 1.6% | 0.045 |
| LRP1B | 8, 17.4% | 43, 34.1% | 0.038 |
| MAP3K13 | 6, 13.0% | 3, 2.4% | 0.012 |

| | | | |
|---|---|---|---|
| NF2 | 4, 8.7% | 0, 0.0% | 0.005 |
| SEMA3E | 5, 10.9% | 2, 1.6% | 0.015 |
| TP53 | 26, 56.5% | 100, 79.4% | 0.006 |
| XRCC3 | 3, 6.5% | 0, 0.0% | 0.018 |
| | Without metastasis (N=144) | With metastasis (N=28) | |
| BRAF | 9, 6.2% | 6, 21.4% | 0.019 |
| EPHA6 | 7, 4.9% | 5, 17.9% | 0.028 |
| GOT1L1 | 0, 0.0% | 2, 7.1% | 0.026 |
| IKZF1 | 1, 0.7% | 3, 10.7% | 0.014 |
| SMARCA1 | 2, 1.4% | 3, 10.7% | 0.031 |
| | Without regional lymph node metastasis (N=93) | With regional lymph node metastasis (N=79) | |
| **ACVR1B** | **9, 9.7%** | **1, 1.3%** | **0.022** |
| ACVR2A | 7, 7.5% | 0, 0.0% | 0.016 |
| APC | 61, 65.6% | 63, 79.8% | 0.042 |
| ARID1A | 19, 20.4% | 6, 7.6% | 0.018 |
| ATRX | 10, 10.8% | 2, 2.5% | 0.039 |
| EPHB4 | 8, 8.6% | 1, 1.3% | 0.040 |
| ETV4 | 0, 0.0% | 7, 8.9% | 0.004 |
| FOXL2 | 0, 0.0% | 4, 5.1% | 0.043 |
| MAP3K13 | 9, 9.7% | 0, 0.0% | 0.004 |
| PIK3CA | 20, 21.5% | 4, 5.1% | 0.002 |
| **PIK3CG** | **7, 7.5%** | **0, 0.0%** | **0.016** |

| | Microsatellite stable (N=165) | Microsatellite instability (N=7) | |
|---|---|---|---|
| ACVR2A | 1, 0.6% | 6, 85.7% | 0.000 |
| ADD3 | 0, 0.0% | 2, 28.6% | 0.001 |
| ADGRA2 | 5, 3.0% | 3, 42.9% | 0.002 |
| ADNP | 1, 0.6% | 2, 28.6% | 0.004 |
| AIM2 | 0, 0.0% | 1, 14.3% | 0.041 |
| AKAP7 | 0, 0.0% | 1, 14.3% | 0.041 |
| ALOX12B | 3, 1.8% | 3, 42.9% | 0.001 |
| ANO10 | 0, 0.0% | 1, 14.3% | 0.041 |
| APC | 122, 73.9% | 2, 28.6% | 0.019 |
| ARID1A | 21, 12.7% | 4, 57.1% | 0.009 |
| ARID1B | 14, 8.5% | 4, 57.1% | 0.002 |
| ARID2 | 10, 6.1% | 4, 57.1% | 0.001 |
| ASH1L | 0, 0.0% | 1, 14.3% | 0.041 |
| ASTE1 | 3, 1.8% | 4, 57.1% | 0.000 |
| ATM | 18, 10.9% | 4, 57.1% | 0.006 |
| ATR | 7, 4.2% | 2, 28.6% | 0.045 |
| ATRX | 9, 5.4% | 3, 42.9% | 0.008 |
| AXIN1 | 0, 0.0% | 2, 28.6% | 0.001 |
| B2M | 3, 1.8% | 2, 28.6% | 0.013 |
| BACH1 | 4, 2.4% | 2, 28.6% | 0.020 |
| BAX | 1, 0.6% | 3, 42.9% | 0.000 |
| BCL2L1 | 0, 0.0% | 1, 14.3% | 0.041 |
| BCL2L2 | 0, 0.0% | 1, 14.3% | 0.041 |

| | | | |
|---|---|---|---|
| BCL6 | 2, 1.2% | 3, 42.9% | 0.000 |
| BCORL1 | 5, 3.0% | 2, 28.6% | 0.027 |
| BMPR2 | 0, 0.0% | 3, 42.9% | 0.000 |
| BORCS8 | 3, 1.8% | 2, 28.6% | 0.013 |
| CARD11 | 15, 9.1% | 5, 71.4% | 0.000 |
| CASP5 | 0, 0.0% | 3, 42.9% | 0.000 |
| CASP8 | 4, 2.4% | 2, 28.6% | 0.020 |
| CCDC168 | 0, 0.0% | 1, 14.3% | 0.041 |
| CCDC43 | 0, 0.0% | 1, 14.3% | 0.041 |
| CD3G | 0, 0.0% | 1, 14.3% | 0.041 |
| CHD4 | 9, 5.4% | 3, 42.9% | 0.008 |
| CLOCK | 0, 0.0% | 2, 28.6% | 0.001 |
| COBLL1 | 0, 0.0% | 2, 28.6% | 0.001 |
| CTCF | 2, 1.2% | 2, 28.6% | 0.008 |
| CTNNA1 | 3, 1.8% | 2, 28.6% | 0.013 |
| CYHR1 | 2, 1.2% | 3, 42.9% | 0.000 |
| DDX27 | 1, 0.6% | 2, 28.6% | 0.004 |
| DOCK3 | 0, 0.0% | 3, 42.9% | 0.000 |
| EGFR | 7, 4.2% | 2, 28.6% | 0.045 |
| EIF2B3 | 1, 0.6% | 2, 28.6% | 0.004 |
| EMSY | 5, 3.0% | 2, 28.6% | 0.027 |
| EP300 | 19, 11.5% | 3, 42.9% | 0.046 |
| EPHA5 | 13, 7.9% | 5, 71.4% | 0.000 |
| EPHB1 | 11, 6.7% | 3, 42.9% | 0.013 |
| EPHB4 | 5, 3.0% | 4, 57.1% | 0.000 |

| | | | |
|---|---|---|---|
| EPHB6 | 15, 9.1% | 3, 42.9% | 0.026 |
| ETV6 | 3, 1.8% | 2, 28.6% | 0.013 |
| FANCL | 3, 1.8% | 2, 28.6% | 0.013 |
| FETUB | 0, 0.0% | 2, 28.6% | 0.001 |
| FGF14 | 2, 1.2% | 2, 28.6% | 0.008 |
| FGF4 | 0, 0.0% | 1, 14.3% | 0.041 |
| FGF6 | 0, 0.0% | 1, 14.3% | 0.041 |
| GBP3 | 1, 0.6% | 2, 28.6% | 0.004 |
| GRK4 | 0, 0.0% | 1, 14.3% | 0.041 |
| HNF1A | 4, 2.4% | 2, 28.6% | 0.020 |
| IGF1 | 0, 0.0% | 1, 14.3% | 0.041 |
| IGF2R | 13, 7.9% | 4, 57.1% | 0.002 |
| IKBKE | 6, 3.6% | 2, 28.6% | 0.035 |
| IKZF1 | 2, 1.2% | 2, 28.6% | 0.008 |
| IL7R | 4, 2.4% | 2, 28.6% | 0.020 |
| IRF4 | 0, 0.0% | 1, 14.3% | 0.041 |
| JAK1 | 4, 2.4% | 2, 28.6% | 0.020 |
| JAK3 | 4, 2.4% | 2, 28.6% | 0.020 |
| JPH4 | 0, 0.0% | 1, 14.3% | 0.041 |
| KCNMA1 | 0, 0.0% | 4, 57.1% | 0.000 |
| KDM5C | 1, 0.6% | 3, 42.9% | 0.000 |
| KDM6A | 5, 3.0% | 2, 28.6% | 0.027 |
| KIT | 10, 6.1% | 3, 42.9% | 0.010 |
| LARP4B | 0, 0.0% | 1, 14.3% | 0.041 |
| LMAN1 | 0, 0.0% | 3, 42.9% | 0.000 |

| | | | |
|---|---|---|---|
| LRP6 | 3, 1.8% | 2, 28.6% | 0.013 |
| LRRIQ3 | 0, 0.0% | 2, 28.6% | 0.001 |
| MAP3K13 | 6, 3.6% | 3, 42.9% | 0.003 |
| MCHR2 | 1, 0.6% | 2, 28.6% | 0.004 |
| MDM2 | 1, 0.6% | 2, 28.6% | 0.004 |
| MED12 | 3, 1.8% | 3, 42.9% | 0.001 |
| MFSD4B | 1, 0.6% | 3, 42.9% | 0.000 |
| MLH1 | 6, 3.6% | 4, 57.1% | 0.000 |
| MRE11 | 1, 0.6% | 2, 28.6% | 0.004 |
| MSH3 | 1, 0.6% | 4, 57.1% | 0.000 |
| MSH6 | 9, 5.4% | 3, 42.9% | 0.008 |
| MYCN | 4, 2.4% | 2, 28.6% | 0.020 |
| NDUFC2 | 1, 0.6% | 2, 28.6% | 0.004 |
| NF1 | 16, 9.7% | 4, 57.1% | 0.004 |
| NOTCH1 | 18, 10.9% | 3, 42.9% | 0.040 |
| NSD1 | 10, 6.1% | 4, 57.1% | 0.001 |
| NSD3 | 2, 1.2% | 3, 42.9% | 0.000 |
| NUP93 | 5, 3.0% | 2, 28.6% | 0.027 |
| OR6C75 | 0, 0.0% | 1, 14.3% | 0.041 |
| PBRM1 | 6, 3.6% | 3, 42.9% | 0.003 |
| PGR | 6, 3.6% | 3, 42.9% | 0.003 |
| PHACTR4 | 0, 0.0% | 1, 14.3% | 0.041 |
| PIK3C3 | 1, 0.6% | 2, 28.6% | 0.004 |
| PIK3CA | 19, 11.5% | 5, 71.4% | 0.001 |
| PRDM2 | 0, 0.0% | 1, 14.3% | 0.041 |

| | | | |
|---|---|---|---|
| PRRG1 | 1, 0.6% | 2, 28.6% | 0.004 |
| PTPRD | 7, 4.2% | 3, 42.9% | 0.004 |
| RAC1 | 0, 0.0% | 1, 14.3% | 0.041 |
| RAD50 | 10, 6.1% | 3, 42.9% | 0.010 |
| RAF1 | 1, 0.6% | 2, 28.6% | 0.004 |
| RB1 | 3, 1.8% | 2, 28.6% | 0.013 |
| RBBP8 | 0, 0.0% | 1, 14.3% | 0.041 |
| RBM27 | 0, 0.0% | 3, 42.9% | 0.000 |
| RBM43 | 0, 0.0% | 1, 14.3% | 0.041 |
| RNF43 | 6, 3.6% | 5, 71.4% | 0.000 |
| ROBO1 | 11, 6.7% | 4, 57.1% | 0.001 |
| ROBO2 | 8, 4.8% | 3, 42.9% | 0.006 |
| RPL22 | 0, 0.0% | 3, 42.9% | 0.000 |
| RUNX1T1 | 5, 3.0% | 2, 28.6% | 0.027 |
| SEC31A | 6, 3.6% | 2, 28.6% | 0.035 |
| SEC63 | 0, 0.0% | 2, 28.6% | 0.001 |
| SETD2 | 7, 4.2% | 3, 42.9% | 0.004 |
| SLC16A4 | 0, 0.0% | 3, 42.9% | 0.000 |
| SLC22A9 | 0, 0.0% | 2, 28.6% | 0.001 |
| SMARCA4 | 4, 2.4% | 5, 71.4% | 0.000 |
| SMC6 | 0, 0.0% | 2, 28.6% | 0.001 |
| SPOUT1 | 0, 0.0% | 1, 14.3% | 0.041 |
| SRPRA | 0, 0.0% | 3, 42.9% | 0.000 |
| TBC1D23 | 0, 0.0% | 1, 14.3% | 0.041 |
| TBX22 | 3, 1.8% | 3, 42.9% | 0.001 |

| | | | |
|---|---|---|---|
| TCERG1 | 0, 0.0% | 1, 14.3% | 0.041 |
| TEAD2 | 0, 0.0% | 2, 28.6% | 0.001 |
| TGFBR2 | 8, 4.8% | 4, 57.1% | 0.000 |
| TM9SF3 | 0, 0.0% | 2, 28.6% | 0.001 |
| TMEM97 | 1, 0.6% | 2, 28.6% | 0.004 |
| TNKS | 8, 4.8% | 6, 85.7% | 0.000 |
| TNKS2 | 6, 3.6% | 3, 42.9% | 0.003 |
| TOP1 | 1, 0.6% | 2, 28.6% | 0.004 |
| TP53 | 124, 75.2% | 2, 28.6% | 0.015 |
| TRRAP | 24, 14.6% | 4, 57.1% | 0.014 |
| TTC3 | 0, 0.0% | 3, 42.9% | 0.000 |
| TTK | 0, 0.0% | 2, 28.6% | 0.001 |
| TVP23A | 0, 0.0% | 2, 28.6% | 0.001 |
| USF3 | 1, 0.6% | 3, 42.9% | 0.000 |
| USP9X | 6, 3.6% | 3, 42.9% | 0.003 |
| VEPH1 | 0, 0.0% | 1, 14.3% | 0.041 |
| VHL | 0, 0.0% | 1, 14.3% | 0.041 |
| WWP1 | 4, 2.4% | 3, 42.9% | 0.001 |
| XPOT | 0, 0.0% | 1, 14.3% | 0.041 |
| ZFR | 0, 0.0% | 1, 14.3% | 0.041 |
| ZNF609 | 0, 0.0% | 1, 14.3% | 0.041 |
| ZNF703 | 3, 1.8% | 2, 28.6% | 0.013 |

| | Without lymphovascular invasion (N=81) | With lymphovascular invasion (N=91) | |
|---|---|---|---|
| ABL2 | 8, 9.9% | 2, 2.2% | 0.048 |

| | | | |
|---|---|---|---|
| DNMT3A | 4, 4.9% | 0, 0.0% | 0.047 |
| INSR | 1, 1.2% | 9, 9.9% | 0.020 |
| MAP3K13 | 8, 9.9% | 1, 1.1% | 0.014 |
| MED12 | 0, 0.0% | 6, 6.6% | 0.030 |
| NF2 | 4, 4.9% | 0, 0.0% | 0.047 |
| NTRK2 | 4, 4.9% | 0, 0.0% | 0.047 |
| PALB2 | 2, 2.5% | 10, 11.0% | 0.036 |
| TP53 | 51, 63.0% | 75, 82.4% | 0.006 |

| | Without diabetes mellitus (N=134) | With diabetes mellitus (N=38) | |
|---|---|---|---|
| ADD3 | 0, 0.0% | 2, 5.3% | 0.048 |
| ARID1B | 10, 7.5% | 8, 21.0% | 0.031 |
| ASTE1 | 3, 2.2% | 4, 10.5% | 0.043 |
| BRD4 | 5, 3.7% | 5, 13.2% | 0.044 |
| CTCF | 1, 0.8% | 3, 7.9% | 0.034 |
| CYHR1 | 1, 0.8% | 4, 10.5% | 0.009 |
| DDX27 | 0, 0.0% | 3, 7.9% | 0.010 |
| ELOA | 0, 0.0% | 2, 5.3% | 0.048 |
| EPHB4 | 4, 3.0% | 5, 13.2% | 0.026 |
| FGF19 | 0, 0.0% | 2, 5.3% | 0.048 |
| GRIN2A | 13, 9.7% | 9, 23.7% | 0.030 |
| H3F3A | 0, 0.0% | 2, 5.3% | 0.048 |
| HRAS | 1, 0.8% | 3, 7.9% | 0.034 |
| KDM5C | 1, 0.8% | 3, 7.9% | 0.034 |
| LRP1B | 46, 34.3% | 5, 13.2% | 0.015 |

| | | | |
|---|---|---|---|
| MBD4 | 0, 0.0% | 2, 5.3% | 0.048 |
| MRE11 | 0, 0.0% | 3, 7.9% | 0.010 |
| NDUFC2 | 0, 0.0% | 3, 7.9% | 0.010 |
| SETD2 | 5, 3.7% | 5, 13.2% | 0.044 |
| SRPRA | 0, 0.0% | 3, 7.9% | 0.010 |
| SUFU | 0, 0.0% | 2, 5.3% | 0.048 |
| TEAD2 | 0, 0.0% | 2, 5.3% | 0.048 |
| TM9SF3 | 0, 0.0% | 2, 5.3% | 0.048 |
| TNKS2 | 2, 1.5% | 7, 18.4% | 0.000 |
| TTK | 0, 0.0% | 2, 5.3% | 0.048 |
| USF3 | 1, 0.8% | 3, 7.9% | 0.034 |

| | BMI ≥ 25 (N=45) | BMI < 25 (N=127) | |
|---|---|---|---|
| ELMO1 | 4, 8.9% | 2, 1.6% | 0.041 |
| GNAQ | 3, 6.7% | 0, 0.0% | 0.017 |
| IRS2 | 4, 8.9% | 2, 1.6% | 0.041 |
| JAK2 | 3, 6.7% | 0, 0.0% | 0.017 |
| RAD50 | 7, 15.6% | 6, 4.7% | 0.042 |
| ROS1 | 8, 17.8% | 8, 6.3% | 0.034 |

**Supplementary Table S3.** Genes that show significantly different mutation frequencies by each clinical information criteria from tumors of the Caucasian patients of the TCGA colorectal cancer data set. Genes in boldface presentation are the common genes that also showed significantly different mutation frequencies from tumors of the 172 Korean patients with colorectal cancer.

| Genes | Categorization by clinical variable | | p-value |
|---|---|---|---|
| | Left-sided (N=70) | Right-sided (N=97) | |
| **APC** | **59, 84.3%** | **59, 60.8%** | **0.001** |
| TRRAP | 2, 2.9% | 18, 18.6% | 0.002 |
| PIK3CG | 0, 0.0% | 13, 13.4% | 0.001 |
| **APCDD1** | **0, 0.0%** | **6, 6.2%** | **0.041** |
| ASXL1 | 3, 4.3% | 14, 14.4% | 0.038 |
| ERBB4 | 4, 5.7% | 17, 17.5% | 0.032 |
| **JAK1** | **0, 0.0%** | **10, 10.3%** | **0.005** |
| ABL2 | 0, 0.0% | 6, 6.2% | 0.041 |
| MSH2 | 0, 0.0% | 9, 9.3% | 0.011 |
| ATR | 1, 1.4% | 9, 9.3% | 0.046 |
| KDR | 0, 0.0% | 6, 6.2% | 0.041 |
| CTNNA1 | 0, 0.0% | 6, 6.2% | 0.041 |
| BRAF | 5, 7.1% | 25, 25.8% | 0.002 |
| WT1 | 1, 1.4% | 12, 12.4% | 0.008 |
| FAT3 | 9, 12.9% | 29, 29.9% | 0.014 |
| FGF23 | 0, 0.0% | 6, 6.2% | 0.041 |

| | | | |
|---|---|---|---|
| ARID2 | 1, 1.4% | 10, 10.3% | 0.026 |
| SH2B3 | 0, 0.0% | 6, 6.2% | 0.041 |
| FANCM | 0, 0.0% | 7, 7.2% | 0.042 |
| BLM | 0, 0.0% | 9, 9.3% | 0.011 |
| NF1 | 0, 0.0% | 16, 16.5% | 0.000 |
| CDK12 | 1, 1.4% | 12, 12.4% | 0.008 |
| RNF43 | 2, 2.9% | 16, 16.5% | 0.005 |
| SOX9 | 4, 5.7% | 17, 17.5% | 0.032 |
| BRD4 | 1, 1.4% | 9, 9.3% | 0.046 |
| ERG | 0, 0.0% | 7, 7.2% | 0.042 |
| BCOR | 0, 0.0% | 15, 15.5% | 0.000 |
| BCORL1 | 1, 1.4% | 13, 13.4% | 0.008 |
| KLHL6 | 1, 1.4% | 10, 10.3% | 0.026 |
| MET | 1, 1.4% | 9, 9.3% | 0.046 |
| MPL | 0, 0.0% | 6, 6.2% | 0.041 |
| PARP1 | 1, 1.4% | 9, 9.3% | 0.046 |
| EPHA7 | 0, 0.0% | 6, 6.2% | 0.041 |
| TNKS | 0, 0.0% | 7, 7.2% | 0.042 |
| WWP1 | 1, 1.4% | 9, 9.3% | 0.046 |
| NKX2-1 | 0, 0.0% | 7, 7.2% | 0.042 |
| CTCF | 1, 1.4% | 9, 9.3% | 0.046 |
| CDH1 | 0, 0.0% | 8, 8.2% | 0.021 |
| FANCA | 0, 0.0% | 13, 13.4% | 0.001 |
| RPTOR | 1, 1.4% | 9, 9.3% | 0.046 |
| AKT2 | 0, 0.0% | 6, 6.2% | 0.041 |

| | | | |
|---|---|---|---|
| CIC | 3, 4.3% | 15, 15.5% | 0.024 |
| ADGRA2 | 0, 0.0% | 7, 7.2% | 0.042 |
| CHD1 | 1, 1.4% | 9, 9.3% | 0.046 |
| ALOX12B | 1, 1.4% | 9, 9.3% | 0.046 |
| IKBKE | 0, 0.0% | 7, 7.2% | 0.042 |
| DNMT3A | 1, 1.4% | 10, 10.3% | 0.026 |
| BAP1 | 0, 0.0% | 6, 6.2% | 0.041 |
| PBRM1 | 1, 1.4% | 10, 10.3% | 0.026 |
| RICTOR | 0, 0.0% | 6, 6.2% | 0.041 |
| DAXX | 0, 0.0% | 8, 8.2% | 0.021 |
| SEMA3E | 1, 1.4% | 9, 9.3% | 0.046 |
| PTCH1 | 1, 1.4% | 14, 14.4% | 0.004 |
| IRS2 | 0, 0.0% | 6, 6.2% | 0.041 |
| ABL1 | 0, 0.0% | 8, 8.2% | 0.021 |
| EWSR1 | 0, 0.0% | 7, 7.2% | 0.042 |
| **INSR** | **0, 0.0%** | **8, 8.2%** | **0.021** |
| SLIT2 | 1, 1.4% | 11, 11.3% | 0.015 |
| **HNF1A** | **0, 0.0%** | **10, 10.3%** | **0.005** |

| | ages under 50 (N=28) | ages 50 or older (N=179) | |
|---|---|---|---|
| APC | 15, 53.6% | 132, 73.7% | 0.042 |
| ERBB4 | 0, 0.0% | 25, 14.0% | 0.030 |
| HOXA3 | 4, 14.3% | 7, 3.9% | 0.045 |
| SMAD4 | 0, 0.0% | 27, 15.1% | 0.030 |
| NCOA3 | 3, 10.7% | 3, 1.7% | 0.034 |

| | | | |
|---|---|---|---|
| RAF1 | 3, 10.7% | 3, 1.7% | 0.034 |
| CHD2 | 4, 14.3% | 7, 3.9% | 0.045 |
| CCNE1 | 2, 7.1% | 1, 0.6% | 0.049 |
| | Low T stage(T1, T2) (N=40) | High T stage(T3, T4) (N=167) | |
| ABL1 | 5, 12.5% | 6, 3.6% | 0.040 |
| ARFRP1 | 3, 7.5% | 1, 0.6% | 0.023 |
| | Without regional lymph node metastasis (N=116) | With regional lymph node metastasis (N=91) | |
| TRRAP | 23, 19.8% | 3, 3.3% | 0.000 |
| **PIK3CG** | **14, 12.1%** | **2, 2.2%** | **0.008** |
| GATA3 | 9, 7.8% | 1, 1.1% | 0.045 |
| ASXL1 | 16, 13.8% | 3, 3.3% | 0.013 |
| PDGFRA | 11, 9.5% | 2, 2.2% | 0.042 |
| TBX3 | 13, 11.2% | 2, 2.2% | 0.014 |
| CDK12 | 14, 12.1% | 1, 1.1% | 0.002 |
| BRD4 | 11, 9.5% | 2, 2.2% | 0.042 |
| FOXL2 | 6, 5.2% | 0, 0.0% | 0.036 |
| CSF1R | 9, 7.8% | 1, 1.1% | 0.045 |
| ETV1 | 8, 6.9% | 0, 0.0% | 0.010 |
| FGFR2 | 10, 8.6% | 1, 1.1% | 0.025 |
| ERCC2 | 6, 5.2% | 0, 0.0% | 0.036 |
| **ACVR1B** | **10, 8.6%** | **1, 1.1%** | **0.025** |
| FANCI | 6, 5.2% | 0, 0.0% | 0.036 |

| | | | |
|---|---|---|---|
| PKHD1 | 16, 13.8% | 3, 3.3% | 0.013 |
| CIC | 18, 15.5% | 3, 3.3% | 0.004 |
| EPHA5 | 16, 13.8% | 4, 4.4% | 0.031 |
| ALOX12B | 10, 8.6% | 1, 1.1% | 0.025 |
| BAP1 | 6, 5.2% | 0, 0.0% | 0.036 |
| BCL6 | 7, 6.0% | 0, 0.0% | 0.019 |
| DAXX | 8, 6.9% | 0, 0.0% | 0.010 |
| PTCH1 | 15, 12.9% | 3, 3.3% | 0.023 |
| KDM5A | 9, 7.8% | 1, 1.1% | 0.045 |
| FAM46C | 9, 7.8% | 1, 1.1% | 0.045 |
| EPHA6 | 16, 13.8% | 3, 3.3% | 0.013 |
| GNAQ | 6, 5.2% | 0, 0.0% | 0.036 |
| TNKS2 | 10, 8.6% | 1, 1.1% | 0.025 |
| CDH5 | 8, 6.9% | 0, 0.0% | 0.010 |
| HNF1A | 10, 8.6% | 1, 1.1% | 0.025 |

| | BMI ≥ 25 (N=113) | BMI < 25 (N=52) | |
|---|---|---|---|
| APCDD1 | 0, 0.0% | 3, 5.8% | 0.030 |
| RAD54L | 1, 0.9% | 4, 7.7% | 0.035 |
| SRSF7 | 0, 0.0% | 3, 5.8% | 0.030 |
| SRC | 1, 0.9% | 4, 7.7% | 0.035 |
| BCORL1 | 4, 3.5% | 8, 15.4% | 0.010 |
| RPTOR | 5, 4.4% | 8, 15.4% | 0.026 |
| PBRM1 | 3, 2.6% | 6, 11.5% | 0.029 |
| NOTCH4 | 3, 2.6% | 8, 15.4% | 0.005 |

| | | | |
|---|---|---|---|
| IRS2 | 1, 0.9% | 4, 7.7% | 0.035 |
| AKT1 | 3, 2.6% | 6, 11.5% | 0.029 |
| GNA11 | 0, 0.0% | 4, 7.7% | 0.009 |
| ITK | 0, 0.0% | 3, 5.8% | 0.030 |
| AXL | 1, 0.9% | 7, 13.5% | 0.001 |
| MEN1 | 0, 0.0% | 4, 7.7% | 0.009 |
| CD79B | 0, 0.0% | 3, 5.8% | 0.030 |
